# Surgically assisted rapid maxillary expasion: a preliminar study

**DOI:** 10.1016/S1808-8694(15)30990-3

**Published:** 2015-10-19

**Authors:** Belmiro Cavalcanti do Egito Vasconcelos, Antonio Figueiredo Caubi, Emanuel Dias, Carlos Augusto Lago, Gabriela Granja Porto

**Affiliations:** a Doctor, Coordinator of the UPE Graduate Program; b Assistant Professor of the Bucomaxillofacial Surgery and Traumatology Department; c Full Professor of the Bucomaxillofacial Surgery and Traumatology Department; d Assistant Professor of the Bucomaxillofacial Surgery and Traumatology Department; e Specialist in Bucomaxillofacial Surgery and Traumatology, Graduate Student of the Master's Degree Course on Bucomaxillofacial Surgery and Traumatology. Pernambuco Dental School - Pernambuco University

**Keywords:** maxillofacial development, maxillary expansion, malocclusion, maxilla/surgery

## Abstract

Surgically assisted rapid maxillary expansion is efficient for the treatment of transverse maxillary deficiencies in skeletally mature patients.

**Aim:**

To study two techniques for surgically assisted rapid maxillary expansion: with or without pterygoid plate detachment.

**Material and Methods:**

A longitudinal cohort study sample including ten patients aged 18-40 years, with a skeletal transverse discrepancy in the maxilla of more than 4 mm. Two groups were established on a randomized basis, five patients in each group, according to the detachment or absence of detachment of the pterygoid plate. Furthermore, osteotomies of the bilateral zygomatic buttress and the intermaxillary suture were done in both groups. The transverse discrepancy was measured in study models, a posterior-anterior cephalometric radiograph evaluated the superior and inferior zygomatic plane and the inter-tuber distance and an occlusal radiograph evaluated the intermaxillary dysjunction in the pre-operative period and 30 days post-operatively. A 7-day period of rest was given after corticotomy before starting expansion with quarter turns once a day.

**Results:**

There were no statistically significant differences between pre- and post-operative measurements.

**Conclusion:**

There are few randomized control trials in literature comparing the two techniques for surgically maxillary expansion. Further studies with a larger sample are required.

## INTRODUCTION

The treatment of malocclusion in adults is frequently difficult because of maxillary defficiencies^1^. Adequate management of transverse deficiencies requires careful pre-treatment to obtain stable and satisfactory occlusion^1^. Proffit et al.^2^ reported that 30% of adult patients that seek orthodontic treatment to correct dentofacial deformities have transverse maxillary deficiencies. The treatment of this conditions during growth is done with orthodontic/orthopedic devices to help separate the palatal suture and other associated structures^3^. Most of the clinical failures in rapid maxillary expansion (RME) using only an orthodontic device are due to pain and resistance to expansion. Recurrence is a significant issue. Furthermore, this palatal expansion technique is inadequate for skeletally mature patients; in these cases surgery becomes necessary^3^.

An alternative to RME in adults is type Le Fort I segmented maxillary osteotomy. However, the morbidity of this procedure is considerably higher than the Le Fort I osteotomy of a single segment. Surgically assisted rapid maxillary expansion (SARME) is an efficient method to treat maxillary deficiencies in skeletally mature patients, having a lower morbidity compared to the Le Fort I procedures mentioned above^4^, ^5^. This treatment is a combination of orthodontic and surgical procedures that increase space in the dental arch and align the teeth^4^. The procedure may include bilateral osteotomy of the zygomatic pillars and the palatal suture with or without separation of the pterygoid processes. The aim of this paper was to study two SARME techniques: the first one included only osteotomy of the zygomatic pillars and the palate, and the second procedure added separation of the pterygoid processes.

## MATERIAL AND METHODS

A pilot study was undertaken between November 2003 and October 2004 at the Bucomaxillofacial Surgery and Traumatology Unit of the Pernambuco University in Recife-PE, Brazil. The study was approved by the university Research Ethics Committee and each patient signed a free and informed consent form.

The sample included ten patients (5 men and 5 women) aged between 18 and 40 years, presenting a transverse maxillary discrepancy over 4mm. Only patients classified as ASA I according to the American Society of Anesthesiologists^6^ and with no history of systemic disease were included in this study.

**Figure 1:**
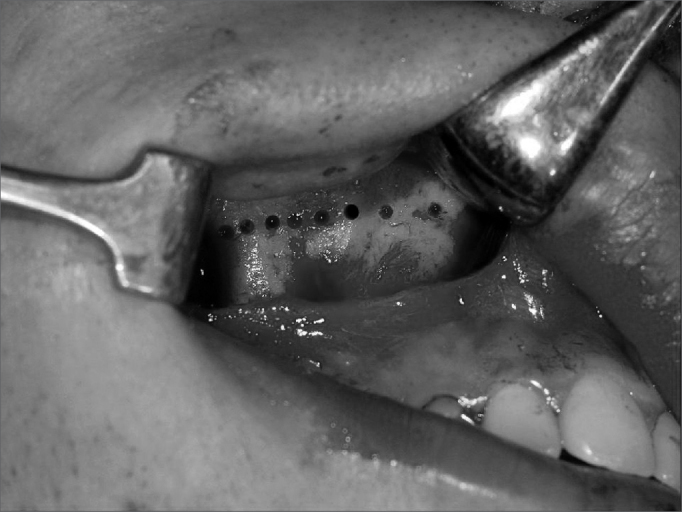
Osteotomy of the anterior and lateral maxillary walls.

**Figure 2:**
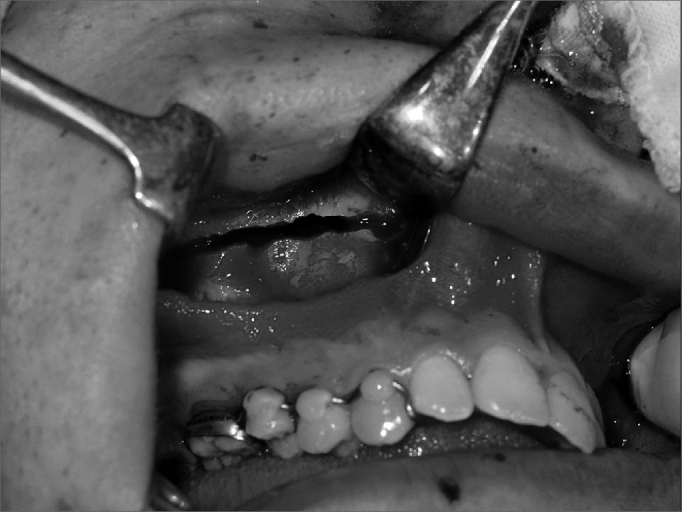
Osteotomy of the anterior and lateral maxillary walls.

**Figure 3:**
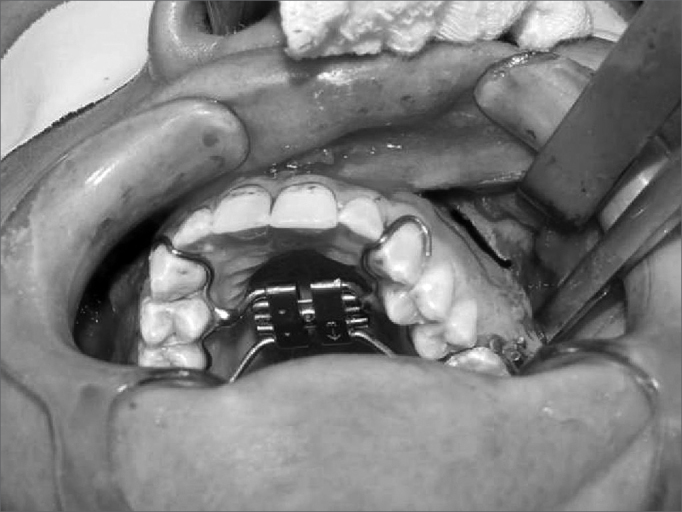
Separation of the pterygoid process.

**Figure 4:**
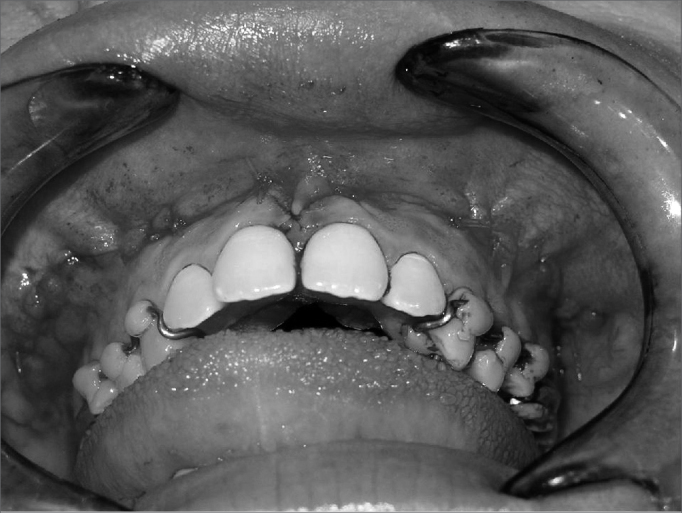
Immediate postoperative.

Surgeries were done by one surgeon. Two five-patient groups were defined randomly, according to whether the pterygoid process was to be separated or not. Group A (control) patients underwent lateral and anterior maxillary wall and intermaxillary suture osteotomies. Group B (experimental) patients underwent similar osteotomies to which was added separation of the pterygoid processes (Figures 1, 2, 3 and 4). The transverse discrepancy was measured in study models as the distance between the upper molar mesiobuccal cusps and the distance between the upper canines preoperatively and 30 days post-operatively. A 0.02 mm precision pachymeter was used for these measurements.

Preoperative and 30-day postoperative anteroposterior cephalometric radiograms assessed the upper and lower zygomatic planes and the intertuberal distance. An occlusal radiogram assessed the intermaxillary disjunction preoperatively and at 30 days postoperatively. Surgery was done under general anesthesia and associated local anesthesia with a vasoconstrictor solution in the labiobuccal vestibule. A horizontal mucoperiosteal incision was made 3mm above the mucogingival junction from the canine to the second molar tooth. A horizontal osteotomy was made on the lateral maxillary wall 4 to 5mm above the tooth apex on level with the occlusal plane and extended from the lateroinferior region of the piriform opening to the zygomatic pillar. The pterygoid process was separated or not according to the group of patients. The intermaxillary region was separated between the central incisors with a fine osteotome. The surgeon placed his second finger over the incisor papillary region to feel the surgical tool as it was passed through the palatine suture. This same surgical tool was also positioned and manipulated in the interradicular region of the central incisor to obtain symmetric maxillary mobilization. An orthodontic device (Hyrax) was activated at 1mm. No expansion was made during the first seven days postoperatively in both groups. After this initial period, patients applied a quarter-turn once a day until the planned expansion was reached.

## RESULTS

**Chart 1:**
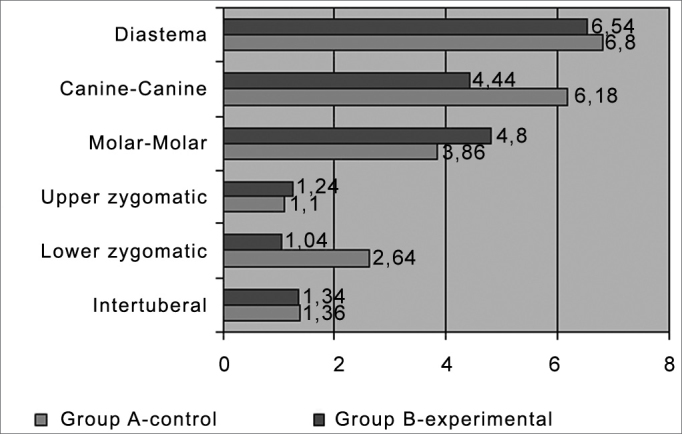
Average differences between preoperative and postoperative measurements in each group.

Table 1 and Chart 1 show the average, the mean and the standard deviation of the preoperative and 30-day postoperative periods for each variable in group A (control) and group B (experimental), and the average differences of the preoperative and postoperative measurements for each group.

**Table 1:** Preoperative and postoperative differences (in mm) of diastema, canine-canine, molar-molar, upper and lower zygomatic and intertuberal planes in each group.

		Group		Total group	
		A-control	B-experi.		
Diastema	Average	6,54	6,80	6,67	0,7775
	Mean	7,50	7,00	7,00	
	Standard deviation	1,80	0,84	1,33	

Canine-Canine	Average	4,44	6,18	5,31	0,3244

	Mean	4,00	5,60	5,05	
	Standard deviation	2,08	3,07	2,63	

Molar-Molar	Average	4,80	3,86	4,33	0,6379

	Mean	5,00	2,50	3,50	
	Standard deviation	2,93	3,15	2,91	

Upper zygomatic	Average	1,24	1,10	1,17	0,8600

	Mean	1,00	1,00	1,00	
	Standard deviation	1,59	0,66	1,15	

Lower zygomatic	Average	1,04	2,64	1,84	0,3707

	Mean	1,00	1,00	1,00	
	Standard deviation	0,95	3,48	2,55	

Intertuberal	Average	1,34	1,36	1,35	0,9739

	Mean	1,00	1,50	1,25	
	Standard deviation	1,15	0,65	0,88

## DISCUSSION

Areas of resistance in the maxilla are the piriform opening (anterior), the zygomatic pillar (lateral), the pterygoid junction (posterior) and the palatine suture (median)^4^. Although the pterygoid processes are considered resistance sites, some surgeons prefer not to separate them due to the risk of injury of the pterygoid plexus by the osteotomy^4^. In our study patients all the areas were osteotomized, except for the pterygoid processes, which were separated or not according to each group.

In this preliminary study there were no significant differences in posterior expansion between both groups, in disagreement with Bays and Greco^7^ who reported that careful separation of the pterygoid processes lead to increased posterior expansion. When these processes are not separated there is a greater tendency for anterior expansion and proportionally less posterior expansion^7^, which was not seen in the control group, in which the preoperative and postoperative differences for anterior and posterior measurements were similar.

In a metanalysis done by Rea et al.^8^ the average maxillary expansion obtained during the postoperative activation phase was 3.99 ± 2.08mm measured at the canines and 6.11 ± 2.64mm measured at the first molar. These measurements are similar to those obtained in our study.

The SARME technique is based on studies by Ilizarov^4^, in which expansion is only started after 5 to 7 postoperatively. This period allows the formation of an initial bone callus but not enough time for bone consolidation. In our study the waiting period before expansion was 7 days.

Surgical complications described in literature include excessive bleeding, maxillary nerve branch injury, infection, pain, devitalization of upper teeth, gingival recession, recurrence, and unilateral expansion^4^, ^9^, ^10^. Complications may increase due to the device used, including breakage or loss of the device, breakage or locking of the fastener, and excessive force over the mucosa leading to necrosis^10^, ^11^. Although pterygoid process separation may increase the risk of injury to the descending palatine artery^12^, ^13^, there was no postoperative bleeding in this study. Both groups had none of the postoperative complications mentioned above.

## CONCLUSION

There are few published randomized studies comparing the techniques of surgically assisted rapid maxillary expansion with and without pterygoid process separation. Although there are many published papers on techniques to correct transverse maxillary hypoplasia, many questions remain unanswered. Given the small sample in this preliminary study, it is not possible to generalize the result that there was no significant difference between both techniques in our sample. Future studies using a similar methodology with larger samples are required to investigate issues such as the recurrence rate, the need to expand beyond the desired end result, possible recurrence factors such as the consolidation time, the total length obtained, intrinsic growth disorders which might cause relapse, the best technique to use, and the type of distractor to use.
